# Toxicity impacts of water treatment sludge disposal in rivers

**DOI:** 10.1007/s10661-026-15557-x

**Published:** 2026-06-25

**Authors:** Marcos Vinicius Mateus, Vinícius Carvalho Rocha, Mário Sérgio da Luz, Rhainer Guillermo Ferreira, Carla Eloísa Diniz dos Santos, Julio Cesar de Souza Inácio Gonçalves

**Affiliations:** Postgraduate Program in Environmental Science and Technology (PPGCTA), Federal University of Triângulo Mineiro (UFTM), Avenida Dr. Randolfo Borges Júnior, N° 1400, Uberaba MG, 38064-200 Brazil

**Keywords:** Aluminum toxicity, Biotic ligand model (BLM), Pollutant dispersion modeling, Brazil, Sludge disposal

## Abstract

Water treatment plants (WTPs) generate sludge rich in aluminum, iron, and suspended solids, which poses environmental risks when improperly disposed. We evaluated the impact of full-cycle WTP sludge discharge into the Uberaba River (Minas Gerais, Brazil), where a fish mortality event occurred downstream. Monitoring campaigns showed extremely high aluminum (11,076 mg/kg) and solid concentrations in the sludge, with downstream aluminum rising from 0.7 to 4.71 mg/L and peaking at 1661 mg/L during temporal sampling. A one-dimensional advection–dispersion model predicted aluminum concentrations up to 360 mg/L under low-flow conditions, exceeding values associated with fish mortality. Biotic ligand model results indicated toxic aluminum binding to gill receptors during peak discharge. Our findings provide evidence linking the sludge disposal to severe water-quality deterioration and the fish kill, while also demonstrating noncompliance with Brazilian environmental regulations. This study underscores the need for regulated sludge treatment and disposal practices to protect aquatic ecosystems.

**Graphical Abstract:**

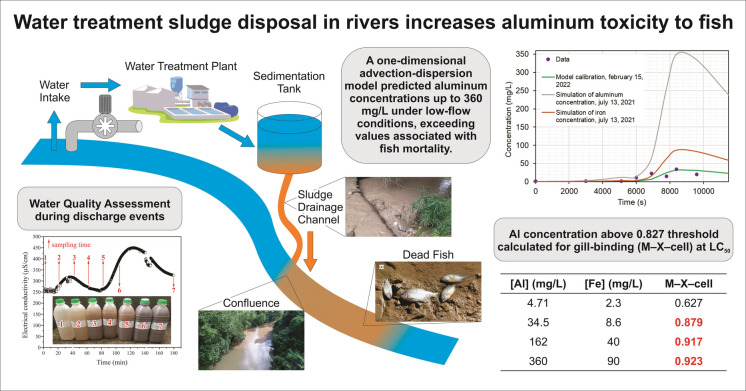

**Supplementary Information:**

The online version contains supplementary material available at 10.1007/s10661-026-15557-x.

## Introduction

A significant source of anthropogenic impact on aquatic systems is the discharge of waste from water infrastructure, including the residual sludge generated by water treatment plants (WTPs). WTPs produce this sludge as a by-product of purifying surface water for potable supplies. If not treated or disposed of adequately, WTP sludge can become an increasing concern due to limitations and lack of water treatment infrastructure (Ahmad et al., 2016; Ahmad, Ahmad, Alam 2016). This sludge contains mineral particles (with diameters less than 2 mm), organic matter, and chemicals from coagulants and polymers (Ahmad et al., 2016), such as aluminum sulfate (Al_2_(SO_3_)_4_·14H_2_O). All of which have a high potential to adversely affect the environment. Long-term inadequate disposal of such sludge in rivers may result in the accumulation of toxic chemicals and metals that can contaminate fish and other animals (Aktar et al., 2011; Batista et al., 2021; Bernegossi et al., 2022).

This issue is particularly relevant in Brazil, where water supply is an essential infrastructure service in basic sanitation systems. Currently, 43% of Brazil rely exclusively on surface water sources, and an additional 14% use a mixed system that is predominantly surface water (ANA, 2021). In other words, surface water supplies 57% of Brazilian cities and serves approximately 156 million inhabitants (ANA, 2021). To comply with drinking water standards, water drawn from surface sources must first be treated at WTPs, where a series of physicochemical processes remove suspended organic matter, pollutants, and microbial contaminants (Ahmad et al., 2016; Pestana et al., 2016). The process often involves adding chemical coagulants like aluminum sulfate, which is particularly notable (Ahmad et al., 2016).

The toxicological risks of aluminum to aquatic life, especially fish, are well-documented. Aluminum is recognized as one of the most acutely harmful metals for freshwater fish due to its capacity to form cationic species that bind strongly to negatively charged sites on fish gills. This binding leads to asphyxiation, impaired ionoregulation, and osmoregulatory collapse (Gensemer & Playle, 1999; Hart et al., 2024). Metals can also induce oxidative stress, disrupt mitochondrial function, and cause metabolic disturbances that compromise survival, feeding, and reproduction (Gashkina, 2024). In aquatic environments, aluminum ions can be absorbed and bioaccumulate in fish tissue (Mert et al., 2014), posing a potential risk to human health through the food chain. Furthermore, its toxicity is often exacerbated in the presence of other metals, such as iron, commonly found in WTP sludge, which increases the metabolic burden on organisms (Dinesh Kumar et al., 2025; Gashkina, 2024). Monitoring data from contaminated rivers worldwide confirm that aluminum concentrations frequently exceed toxicity thresholds for aquatic life (Hart et al., 2024).

Nevertheless, Brazilian facilities commonly dispose sludge in its raw form into water bodies (Batista et al., 2021; Bernegossi et al., 2022; Urban et al., 2018; Wasserman et al., 2018). This practice is largely justified by the belief that, under normal environmental conditions (in terms of pH and redox potential), the sludge is relatively harmless. Indeed, the mineral particulates in the residual sludge are like the natural sediments found on riverbeds. However, many of the chemical coagulants, polymers, and pH-adjusting agents used in water treatment are impure and may contain a variety of contaminants. Some estimate that a WTP treating 1 m^3^/s produces approximately 8300 kg of sludge daily (Frías et al., 2014)—a scenario that can vary with both the quality of the raw water and the operational configuration of the plant (Ahmad et al., 2016; Nayeri & Mousavi, 2022). Despite the known toxicity of aluminum and the scale of sludge generation, quantitative assessments linking the direct disposal of WTP sludge to acute toxic events, specifically through aluminum exposure and fish mortality, remain scarce.

Therefore, this study addressed the case of water treatment sludge disposal in the Uberaba River in Brazil, aiming at measuring and modeling the aluminum levels in the river and its consequence to fish fauna. To reach this goal, the following activities were conducted: (i) sludge characterization from settling tanks in WTPs, (ii) water quality assessment of the Uberaba River during decanter cleaning and filter backwashing operations, and (iii) model development to simulate the impact of sludge discharge on river water quality across distinct hydrological periods.

## Materials and methods

### Study area

**Fig. 1:**
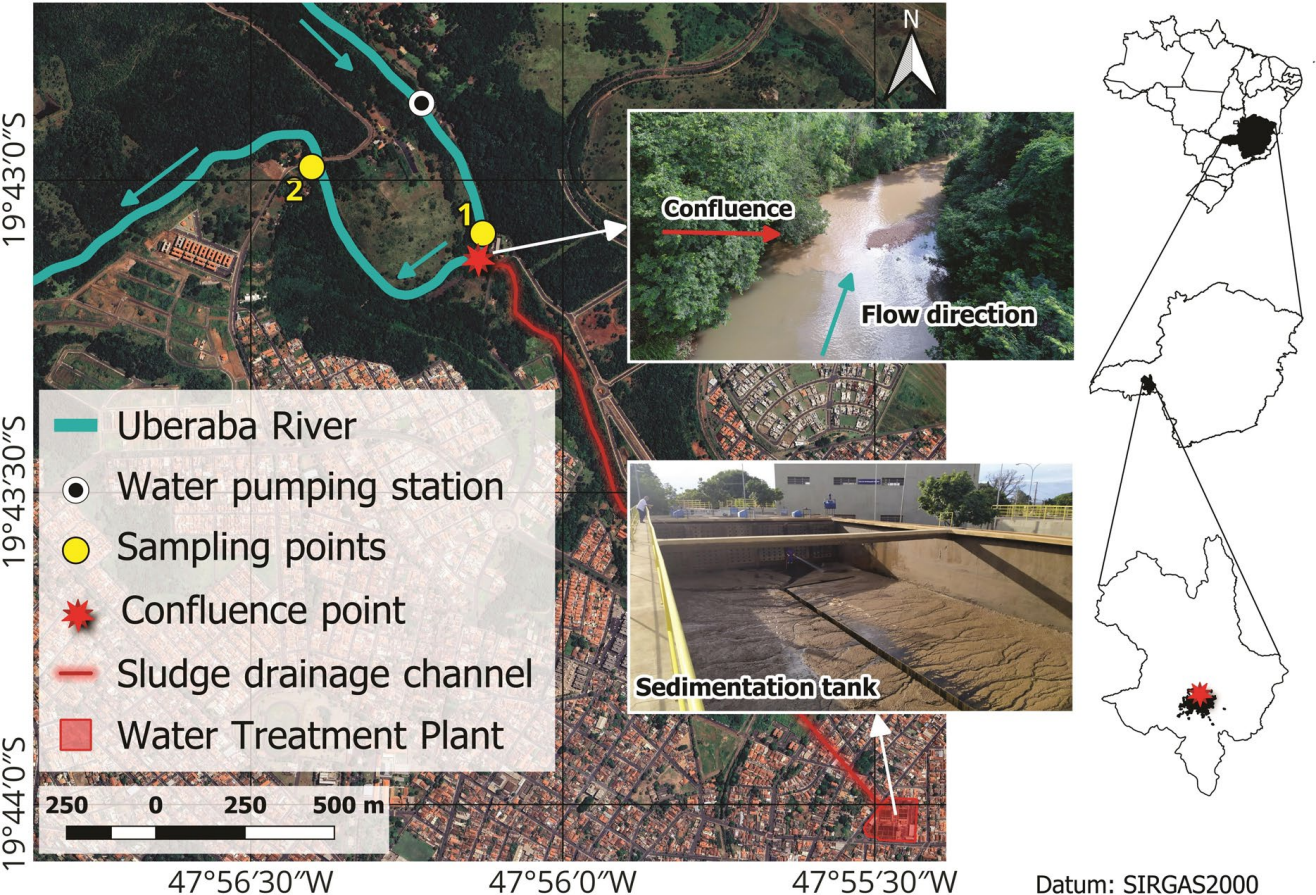
Study area on the Uberaba River, showing the WTP location, the sludge discharge point, and the downstream stretch where fish mortality was observed and sampling was conducted

**Fig. 2:**
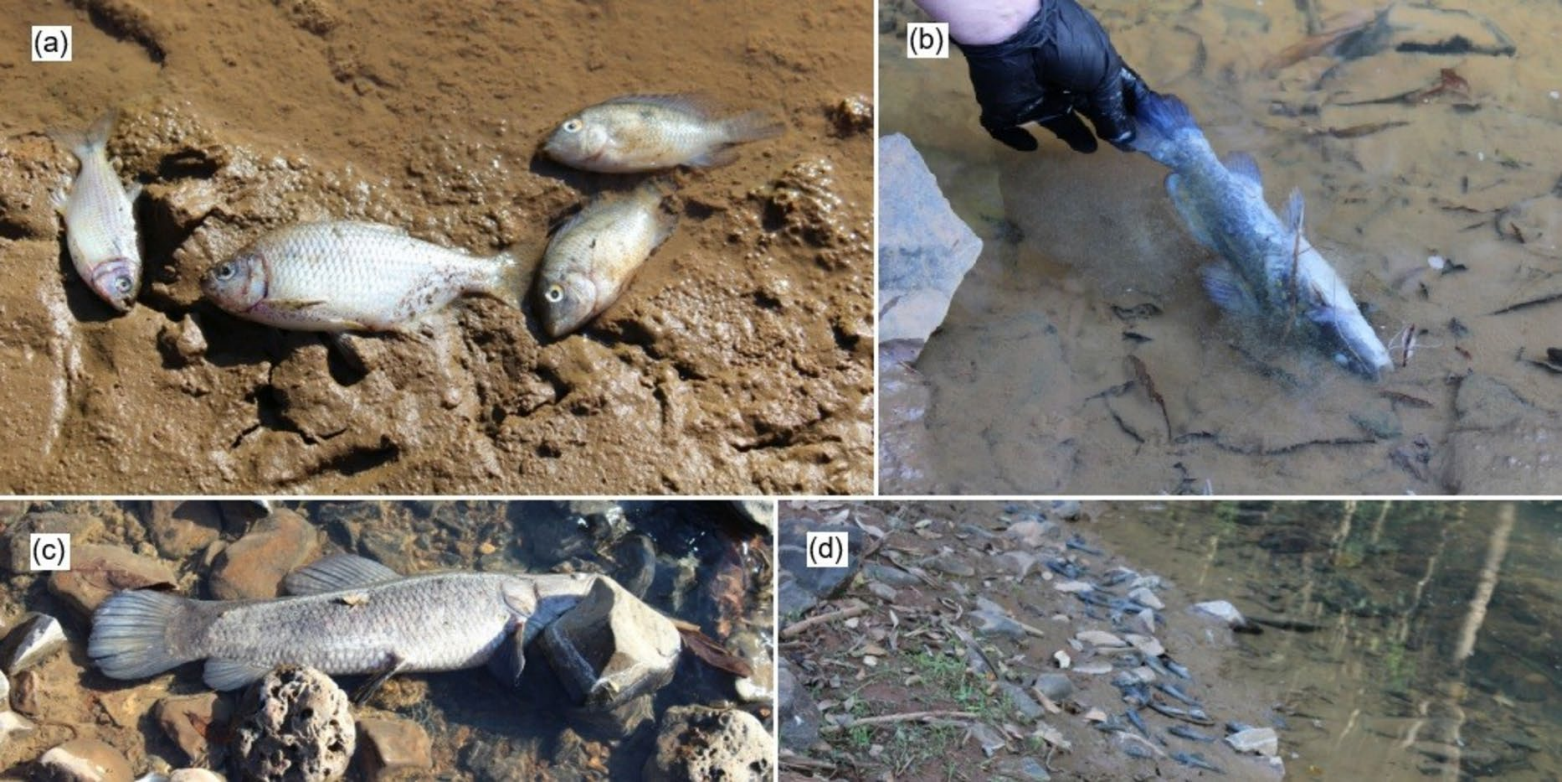
Fish mortality event documented downstream of the WTP sludge discharge point in the Uberaba River. The images show: **a** *Brycon* sp., *Aequidens* sp., *Prochilodus lineatus*; **b** *Rhamdia *sp.;* c* *Hoplias malabaricus*; **d** overview of numerous dead fish along the riverbank following a sludge discharge event

Uberaba is a Brazilian municipality located in the state of Minas Gerais, with an estimated population of approximately 356,781 inhabitants (IBGE, 2025). The main water source for potable supply is the Uberaba River, from which a flow of 1200 L/s is captured (approximately 90% of the total flow). The captured flow is sent to three WTPs, all with full-cycle technology, comprising the stages of coagulation, flocculation, decantation, filtration, and disinfection. Sludge from the decanters and effluent from filter backwashing of WTPs travel approximately 2 km through a drainage system before reaching the Uberaba River at confluence point (19°43′07.60″ S–47°56′08.14″ W), which is indicated as a red star in Fig. 1. On 16 July 2021, a team of officers from the Environmental Military Police identified a significant number of dead fish in the waters of the Uberaba River, downstream from the confluence point with the WTP sludge drainage channel (Fig. 2), prompting this investigation. To assess the impact of waste disposal on the water quality of the Uberaba River, two sampling points were defined: one located 75 m upstream of the discharge point (19°43′05.12″ S–47°56′07.69″ W) and the other 750 m downstream (19°42′58.73″ S–47°56′24.14″ W), as indicated by yellow circles in Fig. 1.

### Water quality and sludge characterization

To directly assess the impact of the sludge discharge, water quality monitoring and sludge characterization were conducted. Sampling campaigns were scheduled to coincide with decanter cleaning procedures and the consequent sludge discharge into the Uberaba River. On 29 November 2021, two river water samples were collected, upstream (19°43′05.12″ S–47°56′07.69″ W) and downstream (19°42′58.73″ S–47°56′24.14″ W) of the confluence point, along with one sludge sample from the WTP sedimentation tank. The analyzed water quality parameters included the following: pH, dissolved oxygen (DO), 5-day biochemical oxygen demand (BOD5), chemical oxygen demand (COD), ammonia nitrogen, total Kjeldahl nitrogen (TKN), settleable solids, total solids, total suspended solids (TSS), total phosphorus, total aluminum, total iron, fixed total solids, and volatile total solids. The samples were collected and analyzed according to Standard Methods for the Examination of Water and Wastewater (APHA, 2017).

Since the WTP residue discharge is transient (occurring only during decanter emptying and washing), a second campaign was designed to capture the temporal dynamics of the pollution plume formed downstream of the discharge point. On 15 February 2022, sampling was performed at different time points in the sludge drainage channel and downstream in the Uberaba River. Parameters analyzed were TDS, settleable solids, total solids, and total aluminum.

**Fig. 3:**
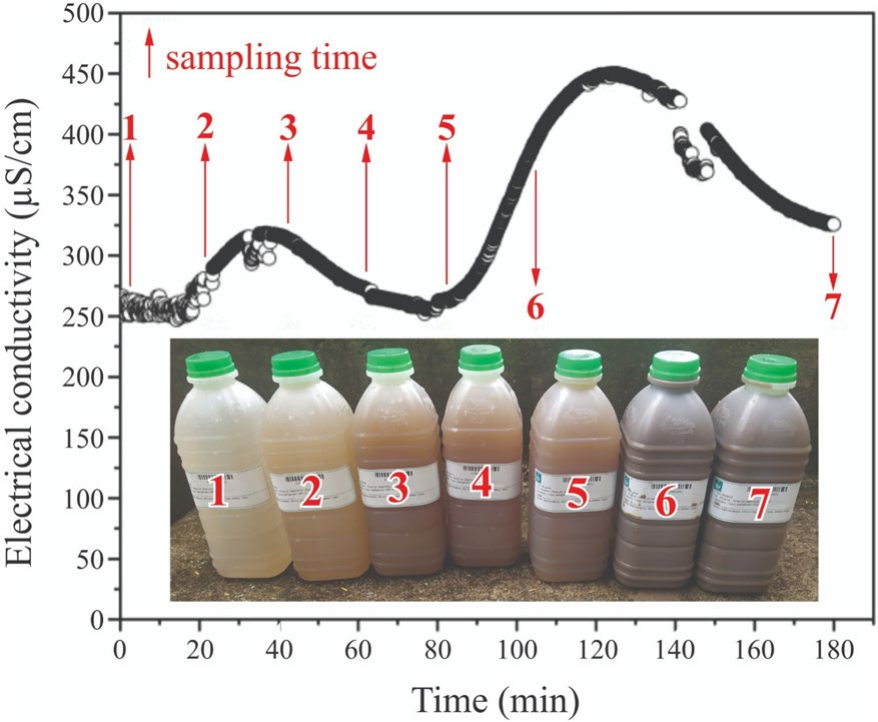
Electrical conductivity profile during sludge passage in the drainage channel. The image within the figure shows the samples collected at different time points

To define the time points for sample collection, ensuring that the entire sludge discharge was monitored, a conductance sensor (Vernier, USA) was installed in the drainage channel to perform readings every 5 s. The resulting electrical conductivity curve during the passage of sludge through the drainage channel and the sampling times are shown in Fig. 3. The sludge passage through the channel lasted 180 min (3 h), presenting two conductivity peaks: the first with a conductivity of 325 μS/cm near minute 35, and the second with a conductivity of 450 μS/cm near minute 120.

Following an initial sample from the sludge drainage channel at 9:18 a.m., the drained sludge was sampled in the river at 10:08 a.m. (50 min later). Subsequent river samples were taken after 35 min (10:43 a.m.) and then at 15-min intervals.

### Pollutant dispersion model in the river

The model used to simulate pollutant concentration is presented in Eq. 1. The first term on the right represents advection, the second represents dispersion, and the third represents the point source of sludge disposal. In this study, the model was employed to simulate aluminum and iron concentrations within the water column. The model construction assumed the absence of physical sinks such as adsorption and sedimentation.

1 $$\frac{dc}{dt}=-U\frac{\partial c}{\partial x}+{D}_{\mathrm{L}}\frac{{\partial }^{2}c}{\partial {c}^{2}}+\frac{W}{V}$$

where *c* represents the aluminum and iron concentration in the river; *U* is the flow velocity; *D*_L_ is the longitudinal dispersion coefficient; *W* is the aluminum load released into the river; and *V* is the volume at the mixing point.

Key model assumptions and inputs were as follows: (I) Aluminum concentration was simulated considering one-dimensional transport (x), assuming complete lateral (y) and vertical (z) mixing; (II) a study reach of 750 m downstream from the discharge (Fig. 1); (III) channel geometry: a prismatic rectangular channel with a mean width of 17 m (estimated using Google Earth Pro 7.3.0.3832); (IV) flow conditions: Two flow scenarios: 7.3 m^3^/s (for model calibration on 15 February 2022) and 0.67 m^3^/s (for simulation of the critical low-flow scenario on 13 July 2021); (V) initial upstream concentrations of 0.7 mg/L Al and 0.1 mg/L Fe; (VI) flow velocity: the mean flow velocity (0.38 m/s) was estimated from the travel time (1980 s) of a pollutant plume between sampling points spaced 750 m apart; cross-sectional area: calculated as the ratio between flow discharge and flow velocity.

The longitudinal dispersion coefficient (*D*_L_) was estimated using the semi-empirical equation from Ribeiro et al. (2010) (Eq. 3). The pollutant load (W) was estimated by multiplying the pollutant concentration in the sludge drainage channel by the flow rate drained during decanter cleaning (W = concentration × flow rate). Since the cleaning flow rate was unknown, it was used as a calibration parameter for the model. Numerical simulation was performed in Vensim PLE (Ventana Systems, Harvard, MA, USA) following the methodology of Gonçalves and Giorgetti (2013), considering a computational element length of 75 m, a time step of 1 s, and a fourth-order Runge-Kutta numerical method.

2 $$D_L=7326U_\ast^{0.303}h^{1.316}b^{0.445}U^{1.458}$$

where U_*_ is the shear velocity (estimated at 0.16 m/s); *h* is the flow depth; and *b* is the flow width.

### Biotic ligand model for toxicity assessment

To link the modeled and measured aluminum concentrations to the observed fish mortality, a biotic ligand model (BLM) was employed. This model estimates the fraction of gill receptors bound by toxic aluminum species, following the approach described by Gensemer and Playle (1999). The binding constant (expressed as logKMBL) for aluminum was considered 13 (Harris & Sheldon, 1990). This value can vary depending on experimental conditions (e.g., pH, ionic strength, and the specific nature of the gill binding sites), but a logKMBL of approximately 13 is a reasonable reference for modeling purposes.

We considered logKFe = 8.3 (Bury & Grosell, 2003), logKAl = 13, and logKH = 7. Hence, we analyzed the competitive binding equation considering the Fe^2^ = 2.3 measured concentration (Table 1). Here we did not consider hardness, but the strong direct competition between iron and aluminum for binding in blood serum (Harris & Sheldon, 1990), to estimate the proportion of receptors bonded to aluminum that would be required for high toxicity (LC50) under 10 mg/L concentrations of aluminum.

**Table 1:** Physicochemical characteristics of water treatment sludge before its disposal (at water treatment plant, WTP), and the river water before (upstream) and after (downstream) sludge reached the Uberaba River

Parameters (unit)	Sludge at WTP	Upstream	Downstream
pH	7.14	6.14	6.10
BOD (mg/L)	13.52	< 2.00	< 2.00
COD (mg/L)	52.27	< 20.00	< 20.00
Sedimented solids	974,090.30 mg/L	< 0.30 mL/L	< 2.0 mL/L
Total solids	10.63%	72.00 mg/L	130.00 mg/L
Total suspended solids	5.20 mg/kg	0.1 mg/L	0.14 mg/L
Ammonia nitrogen (mg/L)	-	< 0.2	< 0.2
Dissolved oxygen (mg/L)	-	7.30	7.40
Kjedahl total nitrogen	266.00 mg/kg	0.84 mg/L	1.68 mg/L
Aluminum	11,076.10 mg/kg	0.7 mg/L	4.71 mg/L
Iron	2,767,12 mg/kg	-	2.30 mg/L
Fixed total solids	9.45%	-	-
Total volatile solids	1.18%	-	-

## Results and discussion

The integrated analysis of field monitoring, numerical modeling, and toxicological assessment provides conclusive evidence that the discharge of untreated WTP sludge into the Uberaba River causes severe aluminum contamination at levels acutely toxic to fish. The results demonstrate a direct link between the sludge disposal practice, the drastic deterioration of water quality during discharge events, and the fish mortality episode, thereby confirming the central hypothesis of this study.

### Physicochemical characterization of the WTP sludge

The results of the physicochemical characterization of the WTP sludge and the assessment of the Uberaba River water quality, upstream and downstream of the confluence point, are presented in Table 1. These data correspond to the initial sampling campaign conducted on 29 November 2021. On this date, the flow rate of the Uberaba River at the study section was 0.70 m^3^/s.

The comparison between upstream and downstream sampling points provides important information about the impact of sludge on the water quality of the Uberaba River. The analysis revealed that parameters including pH, BOD, COD, total phosphorus, ammoniacal nitrogen, and dissolved oxygen showed minimal influence from sludge presence (Table 1). However, a significant increase was observed in water solid fractions after the confluence point. Total solid concentration increased by 81% (Table 1). Table 1 also highlights the notably high aluminum proportion in the sedimentation tank sludge (11,076.10 mg aluminum/kg sludge). Downstream measurements showed an aluminum concentration of 4.71 mg/L, compared to 0.7 mg/L upstream (Table 1). The elevated aluminum concentration in the sludge results from the use of aluminum sulfate as a coagulant in the treatment process, establishing a direct source of this metal to the river.

The characteristics of sludge generated in sedimentation tanks of full-cycle water treatment plants show considerable variability. As demonstrated by Cordeiro (1999) and Di Bernardo et al. (2012), sedimentation tank sludge samples depend primarily on the following: (1) raw water quality, (2) type and dosage of chemicals used, (3) tank cleaning methods, and (4) sludge retention time in the tank. In the WTP examined in this study, the physicochemical characteristics of the sludge vary seasonally, mainly due to changes in raw water quality and consequent adjustments in coagulant dosage. The visual impact of solids reaching the Uberaba River is shown in Fig. 1.

According to Cordeiro (1999), the potential toxicity of WTP sludge for aquatic biota and humans depends on multiple factors, ranging from the sludge characteristics to the hydraulic, chemical, and biological properties of the receiving watercourse. Achon et al. (2005) assessed the environmental impact of WTP sludge discharge using interaction network methodology, which enabled identification of both direct and indirect impacts.

The toxicological effects of aluminum and iron on fish have been increasingly documented in recent scientific literature, especially in the context of metal-rich effluents and acidified aquatic systems. Recent studies emphasize that heavy metals accumulate in fish organs, particularly gills, liver, and intestines, triggering physiological and biochemical disturbances that compromise survival (Dinesh Kumar et al., 2025). Among these metals, aluminum and iron are frequently detected at high concentrations in impacted rivers and exhibit complex modes of toxicity linked to water chemistry, pH, and organic matter levels.

Aluminum is recognized as one of the most acutely harmful metals for freshwater fish due to its capacity to form cationic species such as Al^3^⁺ and Al(OH)^2^⁺ under acidic or low-ionic-strength conditions. These species bind strongly to negatively charged sites on fish gills, leading to asphyxiation, impaired ionoregulation, altered plasma ion balance, and disruptions in nutrient uptake. These mechanisms have been deeply characterized in recent environmental studies, particularly in salmonid species (Hart et al., 2024). The same authors highlight that even relatively low concentrations of inorganic aluminum can exceed toxicity thresholds in sensitive species, causing osmoregulatory collapse and decreased survival of juveniles entering marine waters.

Recent toxicological reviews have also shown that aluminum induces oxidative stress, reduces mitochondrial ATP production, increases energy expenditure for detoxification, and alters metabolic pathways related to hypoxia responses (Gashkina, 2024). These metabolic disturbances compromise swimming performance, feeding behavior, and long-term reproductive fitness. At the cellular level, aluminum triggers elevated generation of reactive oxygen species (ROS), lipid peroxidation, enzyme inhibition, and DNA damage, forming a cascade of metabolic stress that ultimately reduces fitness and survival.

Iron, though essential at trace levels, also becomes toxic at high concentrations, particularly when present in dissolved or colloidal forms capable of entering gill tissues. Accumulation of iron in fish organs has been widely documented in aquatic environments contaminated by industrial and mining effluents, where it disrupts respiratory surfaces, interferes with oxygen uptake, and contributes to oxidative stress (Dinesh Kumar et al., 2025). When combined with aluminum, iron exacerbates the metabolic burden on fish, as simultaneous exposure to multiple metals increases the cellular demand for ATP needed for detoxification, ion regulation, and repair processes (Gashkina, 2024). Such polymetallic exposure has been recognized as one of the major challenges for fish inhabiting chronically contaminated rivers.

Collectively, recent scientific findings demonstrate that aluminum and iron, individually and synergistically, pose significant risks to fish health. These metals impair gill function, disrupt ionic homeostasis, alter mitochondrial energy metabolism, and accumulate in vital organs. Monitoring data from metal-contaminated rivers worldwide further confirms that aluminum concentrations frequently exceed toxicity thresholds for aquatic life (Hart et al., 2024), reinforcing the urgent need for regulatory control of metal-rich effluents. The physiological disruptions observed in contaminated systems are consistent with metabolic stress pathways extensively reviewed in the recent literature (Gashkina, 2024), highlighting that exposure to aluminum and iron compromises ecological integrity and contributes to fish mortality events documented in multiple regions.

In the present study, the primary alterations were associated with solids and metal (aluminum) parameters in the liquid phase. The intensity of these impacts depends on the magnitude of changes in water quality parameters. Therefore, determining the maximum concentrations of solids and aluminum reached in the Uberaba River during sludge discharge events is crucial. To achieve this objective, we conducted additional monitoring, measuring solid fractions and aluminum concentrations throughout the sludge discharge period at three distinct locations: (1) upstream of the confluence point, (2) downstream of the confluence point, and (3) within the sludge drainage channel (Fig. 1). It is worth noting that the literature indicates that metal accumulation in river sediments intensifies environmental risk to aquatic fauna. This is especially true during rainy season floods, when metals are released back into the water due to remobilization process (Slaninova et al., 2014; Sokolov et al., 2023). Nevertheless, in our case study we did not collect sediment samples from the Uberaba River to characterize aluminum and iron presence before and after the event of July 16, 2021. Therefore, in future studies, it is recommended to expand the sampling scope to also evaluate the effect of metals previously present in the river sediment on aquatic fauna.

### Impact of WTP sludge on water quality of the Uberaba River

**Fig. 4:**
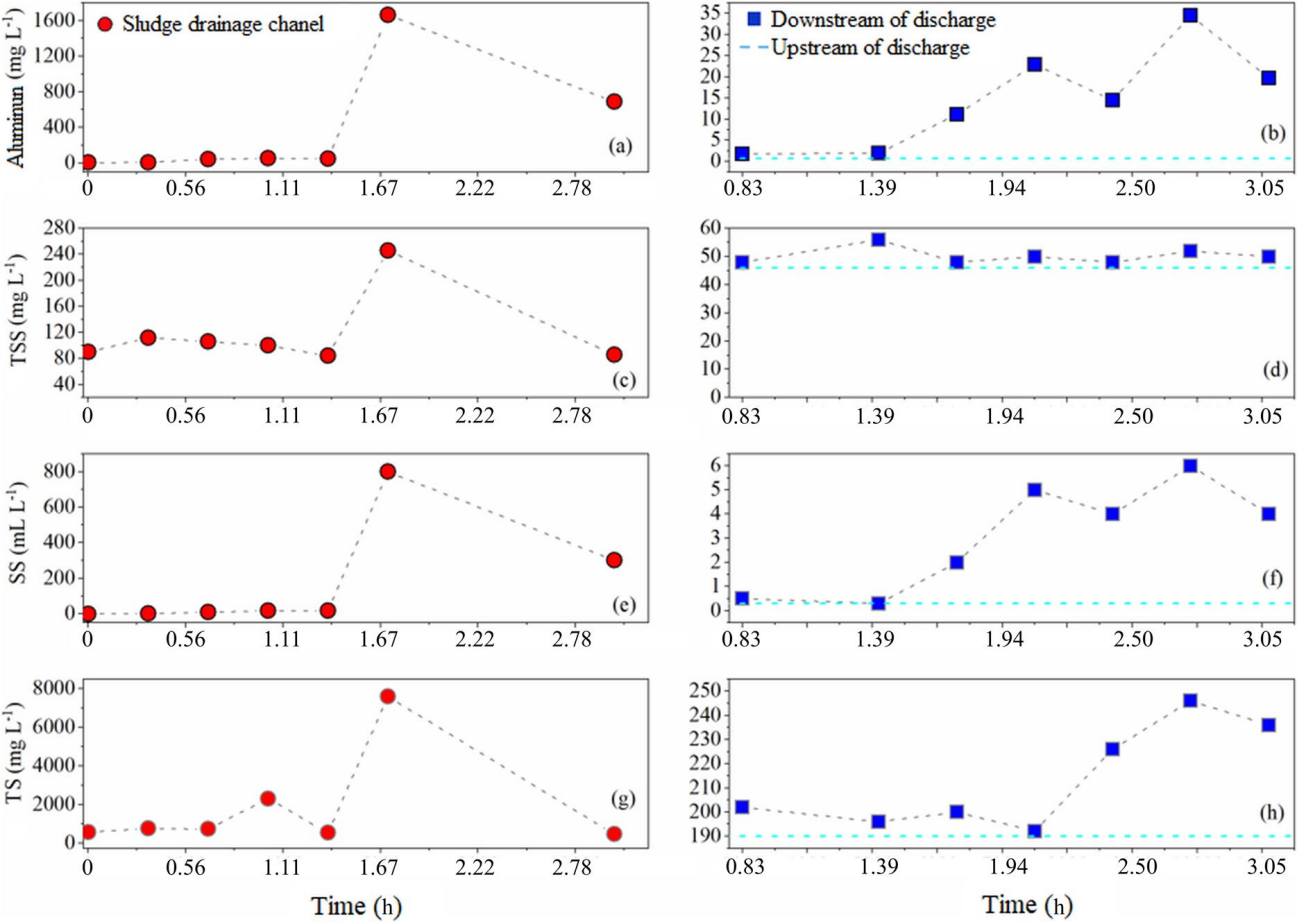
Spatiotemporal dynamics of key pollutants in the Uberaba River during the sludge discharge event

The results from the water quality analyses, for the three points and at the different time intervals, are presented in Fig. 4. It can be observed that the peak pollutant discharge into the Uberaba River occurred at time 6420 s. At this moment, the tank cleaning wash water reached significantly high values of aluminum (1661.35 mg/L), suspended solids (800 mL/L), and total solids (7596 mg/L). The concentration of total suspended solids (TSS) can be estimated by the difference between total solids (TS) and suspended solids (SS). At the peak, the concentration of TSS was 7350 mg/L, a value similar to that reported by Di Bernardo et al. (2012) for wash water from the manual cleaning of conventional sedimentation tanks.

According to NBR-10004 of ABNT (ABNT, 2004), sludges from WTP are classified as solid waste and, therefore, must be treated and disposed of according to the criteria established by this standard. Thus, the direct or indirect discharge of such waste into water bodies is prohibited. If the sludge is considered an effluent—due to the high moisture content—its discharge would still be subject to CONAMA Resolutions No. 357 (Brasil. Conselho Nacional do Meio Ambiente, 2005) and No. 430 (Brasil. Conselho Nacional do Meio Ambiente, 2011), which address the conditions and standards for the disposal of effluents into watercourses.

The CONAMA Resolution No. 430 (Brasil. Conselho Nacional do Meio Ambiente, 2011) establishes that effluents can only be discharged directly into the receiving body if the settleable solids are less than or equal to 1 mL/L. At 6450 s, the suspended solids from the clarifier wash reached 800 mL/L. In the water body, the solids settle and cause negative impacts on the benthic community. According to Silva et al. (2017), the structure of the benthic community downstream of the sludge discharge from WTPs is degraded.

Figure 4b, d, f, and h also show that the water quality of the Uberaba River underwent significant changes after the sludge discharge, as the aluminum concentration increased 49 times at the downstream point (at 10,000 s) compared to the upstream point, and the settleable solids increased 20 times. The flow discharge of the Uberaba River during the discharge event on 15 February 2022 was 7.3 m^3^/s. This is a value 10.4 times greater than the flow recorded during the first inspection on 29 November 2021. Although the Uberaba River had a greater dilution capacity in February, the maximum concentrations of the analyzed parameters were much higher than those in November. This demonstrates that even under higher dilution, the pulse of contamination is severe.

This fact highlights the importance of sampling sludge and the water quality of the Uberaba River at different times. The sampling campaign in November was unable to capture the magnitude of the changes in water quality caused by the sludge discharge.

### Dispersion model calibration and simulation of aluminum and iron concentration in the Uberaba River

**Fig. 5:**
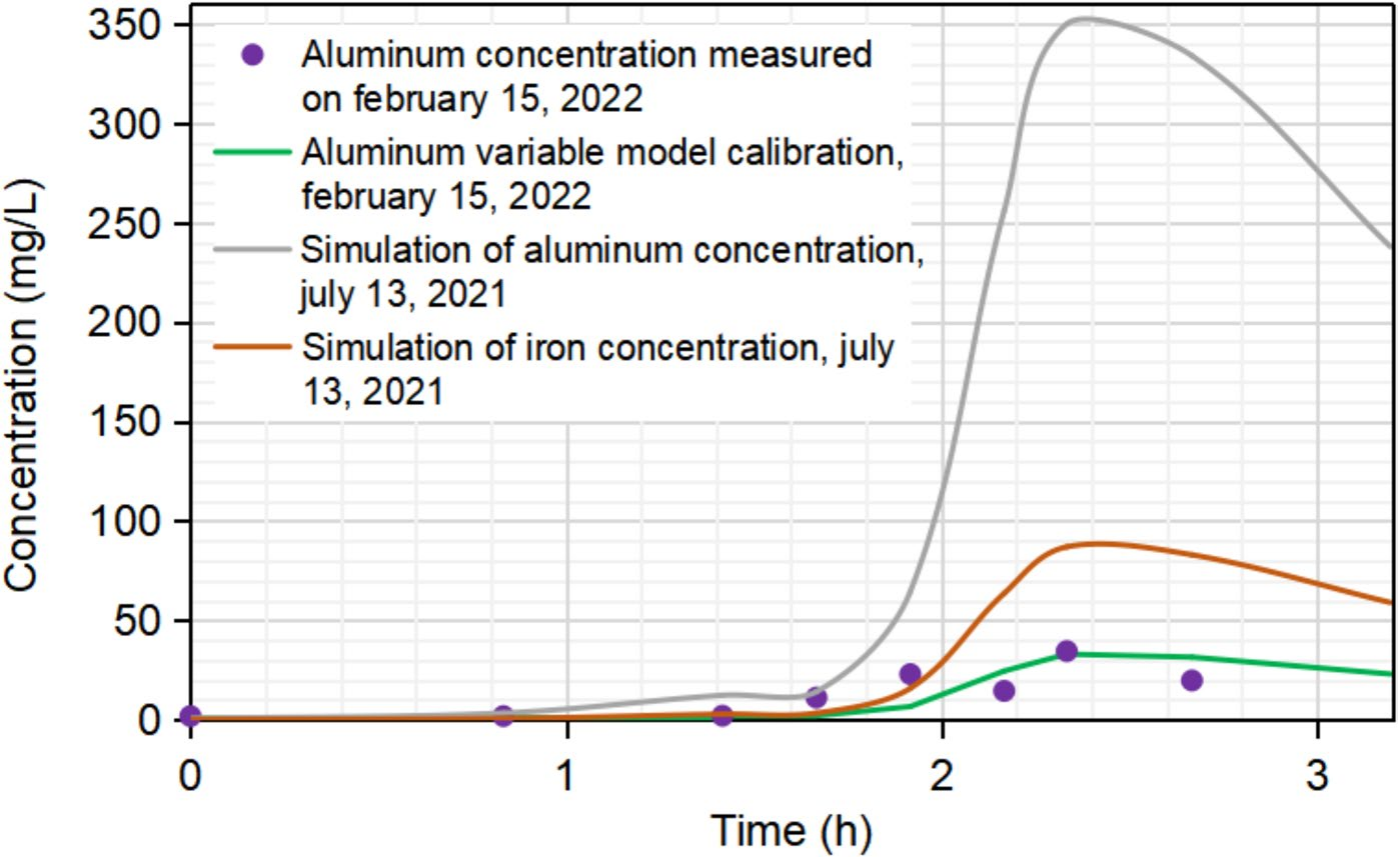
Results of the 1-D advection-dispersion model: calibration against aluminum concentration measured from 15 February 2022 (high flow, 7.3 m^3^/s) and simulation of the critical low-flow scenario (0.67 m^3^/s) representing conditions similar to the July 2021 fish kill event

The simulations are shown in Fig. 5. Given that the sedimentation tank cleaning flow rate was undetermined, it was utilized as a key parameter for model calibration. The calibration process involved adjusting the simulation for 15 February 2022, to match the data collected on the same date. The estimated cleaning flow rate was 0.0975 m^3^/s. Cordeiro (1999) indicates that manual cleaning of sedimentation tanks, using pressurized water jets, typically results in a discharge flow rate of around 0.1 m^3^/s. This value closely aligns with that obtained through model calibration, validating the modeling approach. The comparison between the estimated and measured values was performed using error estimation, namely standard error (SE):

3 $$SE= {\left(\sum\limits_{i=1}^{n}\frac{{\left({c}_{e}-{c}_{m}\right)}^{2}}{n}\right)}^{{~}^{1}\!\left/ \!{~}_{2}\right.}$$

where *n* is the number of measures, *C*_e_ and *c*_m_ are respectively the estimated and measured values of the aluminum concentrations.

The SE obtained in this study was 8.12, a value consistent with other studies simulating water quality and hydrodynamic parameters, as reported by Oliveira et al. (2017).

With the calibrated model, it was possible to reproduce the scenario on 13 July 2021, at which the flow rate of the Uberaba River was 0.67 m^3^/s (Fig. 5). At 8800 s, the aluminum concentration peaked at 360 mg/L (Fig. 5). This concentration exceeds the levels found by Slaninova et al. (2014), who reported fish mortality in Pansky Lake, Czech Republic. Therefore, the model predicts that during the low-flow period coinciding with the reported fish kill, aluminum concentrations in the Uberaba River reached levels known to be lethal.

Slaminova et al. (2014) presented a case study detailing the contamination of Pansky Lake, Czech Republic, in March 2013. An acidic discharge (pH 3.17), characterized by high concentrations of aluminum (119 mg/L) and iron (87 mg/L), caused widespread fish mortality, with affected fish displaying symptoms of asphyxiation. Aluminum and iron content in fish from the impacted lake were found to be 100-fold and 12-fold higher, respectively, compared to fish from a control group (uncontaminated lakes). In fish, aluminum is associated with gill damage, primarily due to its deposition and the subsequent disruption of osmoregulation. Gensemer and Playle (1999) proposed a model describing the respiratory and ionoregulatory effects of aluminum on fish. Respiratory effects result from the precipitation of aluminum on the gills. Conversely, ionoregulatory disturbances are hypothesized to be caused by the interaction between positively charged aluminum species and the negatively charged gill surface. The accumulation of aluminum on the gills leads to a decrease in blood PO_2_ and an increase in blood PCO_2_, which, in conjunction with ionoregulatory effects, can ultimately result in fish mortality.

### Biotic ligand model for toxicity assessment

The BLM was employed to directly assess whether the elevated aluminum concentrations measured and modeled in the Uberaba River were sufficient to trigger the acute toxic mechanisms described in the literature. Results of the BLM, using the baseline values of aluminum and iron in Table 1, the highest results in Fig. 4, and the highest result in the predictive model (Fig. 5), are shown in Table 2. Results show that the sludge disposal causes the increase of the fraction of receptors (e.g., transferrin) bonded to Al, above the LC_50_ fraction of 0.827 (Table 2).

**Table 2:** Biotic ligand model results of Al-gill binding

Aluminum concentration (mg/L)	Iron concentration (mg/L)	M-X-cell
4.71	2.30	0.627
34.53	8.63	0.879*
161.98	40.50	0.917*
360.00	90.00	0.923*

*Above the 0.827 threshold calculated for gill-binding at LC_50_ [Al^3+^] = 10 mg/L

Therefore, the BLM results provide the conclusive mechanistic link between the sludge discharge and the fish kill event. They confirm that the aluminum levels in the Uberaba River during and after sludge disposal were not only high but were specifically bioavailable in forms capable of binding to fish gills at a level known to cause acute ionoregulatory failure, respiratory distress, and ultimately mortality, consistent with the toxicological pathway described by Gensemer and Playle (1999), Mert et al. (2014), Hart et al. (2024), and Gashkina (2024). This analysis confirms that the sludge disposal caused a toxicological impact sufficient to be the most probable cause of death for the fish observed downstream (Fig. 2).

Toxicity of precipitated aluminum on aquatic organisms is a chronic issue not only in water treatment plants, but water pollution in general. It is dependent on pH, with a minimum solubility near pH 6 and increasing at both lower and higher pH values (Santore et al., 2018). Furthermore, other metals and dissolved carbon may compete with aluminum for ligand sites in blood cells. Thus, although high iron concentrations were found in our samples, aluminum precipitation was lethal even though iron can lead to lower toxicity of aluminum. Therefore, it is urgent to create site-specific water guidelines considering local biodiversity and fish species sensitivity, as well as hydrobasin characteristics (Niyogi & Wood, 2004).

### Regulatory framework and legal impediments to WTP sludge discharge

The direct release of sludge from WTPs into natural watercourses constitutes a clear violation of Brazilian environmental law, regardless of its physical characteristics or dilution conditions. Under ABNT NBR 10004, WTP sludge is expressly classified as a solid waste, meaning that its disposal in rivers is legally prohibited. Even when interpreted as an effluent due to its liquid fraction, the material fails to meet the environmental compatibility requirements established by CONAMA Resolutions No. 357 (Brasil. Conselho Nacional do Meio Ambiente, 2005) and No. 430 (Brasil. Conselho Nacional do Meio Ambiente, 2011), which forbid any discharge capable of altering water quality, depositing solids in the aquatic environment, or compromising aquatic life. These regulations underscore that water bodies used for public supply, such as the Uberaba River, must maintain minimum quality standards consistent with Class 2 waters, particularly regarding metals and suspended solids. CONAMA Resolution No. 430/2011 establishes a maximum concentration of settleable solids of 1 mL/L (Brasil. Conselho Nacional do Meio Ambiente, 2011). Furthermore, CONAMA Resolution No. 357/2005 states that the maximum permitted concentration of dissolved aluminum in Class 2 rivers is 0.1 mg/L if the water pH is greater than 5.5 (Brasil. Conselho Nacional do Meio Ambiente, 2011). Therefore, considering the data presented in Table 1, at the point downstream of the residual sludge discharge, both the sedimented solid concentration (2.0 mL/L) and the aluminum concentration (4.71 mg/L) exceeded the legal limits.

In the context of Uberaba, this legal framework becomes even more restrictive due to the river’s essential role as the primary source of drinking water for the municipality. The introduction of WTP sludge into the river, even during temporary sedimentation tank cleaning operations, results in significant changes to turbidity, solid concentration, and metal content. Such alterations violate the fundamental provisions of CONAMA Resolution No. 357 (Brasil. Conselho Nacional do Meio Ambiente, 2005), which requires that effluent discharges must not degrade the quality of receiving waters or impede their designated uses. Moreover, CONAMA Resolution No. 430 (Brasil. Conselho Nacional do Meio Ambiente, 2011) emphasizes the prohibition of discharges that trigger ecological harm, including the impairment of aquatic biota, a concern that becomes critical when considering the episodes of fish mortality documented downstream from the confluence point.

The legal implications extend to the realm of criminal liability under Brasil’s 1998 Environmental Crimes Act (Brasil, 1998). This legislation characterizes as an environmental crime any act that results in the death of aquatic organisms due to the release of pollutants into water bodies. In Uberaba, where the local hydrology is marked by seasonality and periods of low river flow, the river’s reduced dilution capacity amplifies the impacts of pollutant loads. Consequently, the release of sludge rich in metals and solids not only represents an administrative infraction but may also constitute criminal environmental damage, especially when associated with verified harm to aquatic fauna.

Finally, the National Solid Waste Policy (Brazilian law N^o.^ 12.305/2010) establishes fundamental principles for the environmentally appropriate management of solid waste and explicitly prohibits its disposal in bodies of water. The discharge of WTP sludge into the Uberaba River directly contravenes the policy’s guidelines on prevention, precaution, and the protection of water resources. As such, the practice violates multiple legal instruments simultaneously, illustrating a systemic failure in waste management and environmental compliance. The findings of this study—documenting pollutant concentrations, predicting lethal conditions, and confirming toxicological impact—provide the scientific evidence underlying these legal violations. The necessity for proper sludge treatment and disposal practices is highlighted to preserve the ecological integrity of the Uberaba River and safeguard public health.

If the appropriate disposal of residual sludge in a sanitary landfill is not promptly carried out (due to financial, logistical, or other issues), it is also recommended that the companies responsible for WTP operation establish a frequent routine for monitoring river water quality before and after residual sludge discharge. Such a routine is even more necessary during dry periods.

## Conclusion

This study demonstrated that the discharge of sludge into rivers, originating from a WTP sedimentation tank that utilizes aluminum sulfate as a coagulant, can cause mortality in various fish species. The advection–dispersion numerical model was utilized to estimate the concentrations of iron and aluminum present at the time of fish mortality. The maximum concentrations found were 360 mg/L for aluminum and 90 mg/L for iron, respectively. The biotic ligand model (BLM) indicated that an increase in the fraction of Al-bound receptors, exceeding the LC_50_ fraction of 0.827, was the most probable cause of fish mortality. The spatial and temporal sampling effort revealed the dynamics of the solids and chemical plume during WTP sedimentation tank cleaning, and its subsequent impact on water quality and aquatic fauna. Finally, our findings highlight the necessity for proper sludge treatment and disposal practices to preserve the ecological integrity of the Uberaba River and safeguard public health.

## Supplementary Information

Below is the link to the electronic supplementary material.

ESM 1 (DOCX 22.3 KB)

## Data Availability

The input and output data used for computational modeling have been shared as supplementary material to the article. The reports containing the physicochemical characterization of the river and WTP sludge samples, as well as the Vensim software modeling file, are in Portuguese and can be made available upon request to the corresponding author.
