# Supplementary material for: Toxicity impacts of water treatment sludge disposal in rivers
Source: Environ Monit Assess. 2026 Jun 25;198(7):767. doi: 10.1007/s10661-026-15557-x (PMC13303562; doi:10.1007/s10661-026-15557-x)
Supplement: Supplementary file 1 — (DOCX 22.3 KB) [file 10661_2026_15557_MOESM1_ESM.docx]

| **3.1 Physicochemical characterization of the WTP sludge** |
| --- |
| The results of the physicochemical characterization of the WTP sludge and the assessment of the Uberaba River water quality, upstream and downstream of the confluence point, are presented in Table 3.1. These data correspond to the initial sampling campaign conducted on 29 November 2021. On this date, the flow rate of the Uberaba River at the study section was 0.70 m³/s. |
| **Table 3.1** Physicochemical characteristics of water treatment sludge before its disposal (at water treatment plant, WTP), and the river water before (upstream) and after (downstream) sludge reached the Uberaba River.   \| **Parameters (unit)** \| **Sludge at WTP** \| **Upstream** \| **Downstream** \| \| --- \| --- \| --- \| --- \| \| pH \| 7.14 \| 6.14 \| 6.10 \| \| BOD (mg/L) \| 13.52 \| < 2.00 \| < 2.00 \| \| COD (mg/L) \| 52.27 \| < 20.00 \| < 20.00 \| \| Sedimented solids \| 974,090.30 mg/L \| < 0.30 mL/L \| < 2.0 mL/L \| \| Total solids \| 10.63% \| 72.00 mg/L \| 130.00 mg/L \| \| Total suspended solids \| 5.20 mg/Kg \| 0.1 mg/L \| 0.14 mg/L \| \| Ammonia nitrogen (mg/L) \| - \| < 0.2 \| < 0.2 \| \| Dissolved oxygen (mg/L) \| - \| 7.30 \| 7.40 \| \| Kjedahl total nitrogen \| 266.00 mg/Kg \| 0.84 mg/L \| 1.68 mg/L \| \| Aluminum \| 11,076.10 mg/Kg \| 0.7 mg/L \| 4.71 mg/L \| \| Iron \| 2,767,12 mg/kg \| - \| 2.30 mg/L \| \| Fixed total solids \| 9.45 % \| - \| - \| \| Total volatile solids \| 1.18 % \| - \| - \| |

| **3.2 Impact of WTP sludge on water quality of the**  **Uberaba River** | | | | |
| --- | --- | --- | --- | --- |
| The results from the water quality analyses, for the three points and at the different time intervals, are presented in  Table 3.2. | | | | |
| **Table 3.2** Spatio-temporal dynamics of key pollutants in the Uberaba River during the sludge discharge event. | | | | |
| **Downstream of discharge** | | | | |
| **Time** | **Al (mg/L)** | **SDT (mg/L)** | **SS (mL/L)** | **ST (mg/L)** |
| 10h08 | 1.8 | 48 | 0.5 | 202 |
| 10h43 | 1.98 | 56 | 0.3 | 196 |
| 10h58 | 11.14 | 48 | 2 | 200 |
| 11h13 | 22.93 | 50 | 5 | 192 |
| 11h28 | 14.49 | 48 | 4 | 226 |
| 11h38 | 34.53 | 52 | 6 | 246 |
| 11h58 | 19.75 | 50 | 4 | 236 |
| **Sludge drainage channel** | | | | |
| **Time** | **Al (mg/L)** | **SDT (mg/L)** | **SS (mL/L)** | **ST (mg/L)** |
| 9h18 | 6.57 | 90 | 0.5 | 596 |
| 9h38 | 9.63 | 112 | 2 | 774 |
| 10h00 | 44.03 | 106 | 10 | 744 |
| 10h20 | 53.47 | 100 | 18 | 2322 |
| 10h40 | 52.03 | 84 | 18 | 560 |
| 11h05 | 1661.35 | 246 | 800 | 7596 |
| 12h18 | 691.73 | 86 | 300 | 486 |

| **3.3 Dispersion model calibration and simulation of aluminum and iron concentration in**  **the Uberaba River** | | | | |
| --- | --- | --- | --- | --- |
| The model equation used to simulate pollutant concentration and key model assumptions are presented in the main text under section "2.3 Pollutant dispersion model in the river". The  model entry data, calibration and simulation results are presented in Table 3.3. | | | | |
| **Table 3.3** Results of the 1-D advection-dispersion model: calibration against measured data  from 15 February 2022 (high flow, 7.3 m³/s) and simulation of the critical low-flow scenario (0.67 m³/s) representing conditions similar to the July 2021 fish kill event. | | | | |
| **Time (s)** | **Data** | **Model calibration, February 15, 2022** | **Simulation of aluminum concentration,**  **July 13, 2021** | **Simulation of iron concentration,**  **July 13, 2021** |
| 0 | 1.8 | 0.7 | 0.7 | 0.1 |
| 3000 | 1.8 | 0.94 | 3.33 | 0.6 |
| 5100 | 1.98 | 1.8 | 12.3 | 2.8 |
| 6000 | 11.14 | 1.95 | 14.1 | 3.3 |
| 6900 | 22.93 | 6.6 | 64.5 | 15.8 |
| 7800 | 14.49 | 24.5 | 256 | 63.6 |
| 8400 | 34.53 | 33 | 350 | 87 |
| 9600 | 19.75 | 31.5 | 334 | 83 |
| 11500 | - | 23 | 238 | 59 |

| **3.4 Biotic ligand model for toxicity assessment** | | |
| --- | --- | --- |
| The key Biotic Ligant Model (BLM) assumptions are presented in the main text under section "2.4 Biotic ligand model for toxicity assessment". The entry data for the BLM are the baseline values of aluminum and iron (downstream characterization in Table 3.1), the highest results in downastream impact assessment (Table 3.2), and the highest results in the predictive model (Table 3.3). The BLM results (M- X-cell) are presented in Table 3.4. | | |
| **Table 3.4** Biotic ligand model results of Al-gill binding. | | |
| **Aluminum**  **concentration (mg/L)** | **Iron concentration (mg/L)** | **M-X-cell** |
| 4.71 | 2.30 | 0.627 |
| 34.53 | 8.63 | 0.879 |
| 161.98 | 40.50 | 0.917 |
| 360.00 | 90.00 | 0.923 |
